# Microdissection of the Bulk Transcriptome at Single-Cell Resolution Reveals Clinical Significance and Myeloid Cells Heterogeneity in Lung Adenocarcinoma

**DOI:** 10.3389/fimmu.2021.723908

**Published:** 2021-09-30

**Authors:** Hao Wu, Jiale Qin, Qiang Zhao, Lu Lu, Chen Li

**Affiliations:** ^1^ Department of Human Genetics, Department of Ultrasound and Women’s Hospital, Zhejiang University School of Medicine, Hangzhou, China; ^2^ Zhejiang Provincial Key Laboratory of Precision Diagnosis and Therapy for Major Gynecological Diseases, Hangzhou, China; ^3^ M&D Data Science Center, Tokyo Medical and Dental University, Tokyo, Japan

**Keywords:** tumor infiltrating myeloid cells, lung adenocarcinoma, single-cell transcriptome analysis, bulk RNA-seq analysis, deconvolution

## Abstract

**Background:**

Tumor infiltrating myeloid (TIM) cells constitute a vital element of the tumor microenvironment. The cell-type heterogeneity of TIM has yet to be fully investigated.

**Methods:**

We used a time saving approach to generate a single-cell reference matrix, allowing quantification of cell-type proportions and cell-type-specific gene abundances in bulk RNA-seq data.

**Results:**

Two distinct clusters, MSC1 and MSC2 (MSC subtype) were newly identified in lung adenocarcinoma (LUAD) patients, both significantly associated with overall survival and immune blockade therapy responses. Twenty myeloid cell types were detected. Thirteen of these had distinct enrichment patterns between MSC1 and MSC2. LAMP3+ dendritic cells, being a mature and transportable subtype of dendritic cell that may migrate to lymph nodes, were noted as associated with non-responsiveness to immunotargeted therapy. High infiltration level of IFIT3+ neutrophils was strongly related to the response to immune-targeted therapy and was seen to activate CD8+ T cells, partly through inflammasome activation. The infiltration levels of TIMP1+ macrophages and S100A8+ neutrophils were both significantly associated with poor prognosis. TIMP1+ macrophages were noted to recruit S100A8+ neutrophils *via* the CXCL5–CXCR2 axes and promote LUAD progression.

**Conclusion:**

Altogether, we performed virtual microdissection of the bulk transcriptome at single-cell resolution and provided a promising TIM infiltration landscape that may shed new light on the development of immune therapy.

## Introduction

Lung adenocarcinomas (LUADs) account for over 40% of lung cancers and represent its most leading and prevalent histological subtype. Despite an improvement in therapeutic strategies, the rates of objective clinical responses remain low, with only 17.4% lung cancer patients surviving more than 5 years beyond diagnosis (1). In this, the dynamic tumor immune microenvironment plays an important role in tumor progression and metastasis (2, 3). Tumor-infiltrating T lymphocytes are now recognized as the key components of the tumor microenvironment (TME). Therapeutic strategies for targeting these cells are being actively developed and have demonstrated remarkable therapeutic effects (4). While current immunotherapies targeting T lymphocytes benefit only few patients (5), it is important to unravel the exact cellular functions of the remaining cell types within the TME that may be involved in tumor progression.

Recently, there has been much focus on cancer immunology, with primary emphasis on myeloid cells as important components in tumor immune evasion (6, 7). However, the various reports that ascribe macrophages and neutrophils with either pro- or antitumor properties, together with acknowledgement that our understanding of tumor infiltrating myeloid cell (TIM) subtypes is quite inadequate (8, 9), leads to a high potential for confusion and/or contradiction within this field. Despite this, some strategies targeting myeloid cells have been developed (10, 11). However, the limited understanding of clear mechanistic hypotheses had led to difficulties in interpreting clinical outcomes for such approaches (12). In particular, the complexity of TIM subtypes and the discrepancies between human and mouse models has deeply impeded the implementation of selective myeloid-targeting immunotherapies.

Single-cell RNA sequencing (scRNA-seq) offers an opportunity to dissect the complexity of the TME, enabling the identification of the cell state in a manner independent of any previous knowledge of cellular markers (13, 14). Single-cell analysis has been applied to reveal the cellular heterogeneity of TIMs, including tumor-associated macrophages (TAMs), dendritic cells (DCs), and neutrophils in different cancer types (13, 15, 16). However, due to high cost and strict requirement for cellular activity, analyses of large patient cohorts have been almost impossible. Computational algorithms, which allow for the estimation of relative cell infiltrate level based on bulk RNA sequencing (RNA-seq) and scRNA-seq data, have now been developed and may compensate for this (17–19). CIBERSORTx is a computational framework to accurately infer relative abundance of cell type from bulk RNA-seq according to the signature matrix generated from scRNA-seq by means of a deconvolution algorithm (18). It has been successfully used and validated for revealing immune cell landscapes in melanomas (20), clear-cell renal cell carcinomas (21), and prostate cancer (22). Unfortunately, CIBERSORTx does not seem to provide a standard procedure or pipeline describing how to integrate scRNA-seq data to construct a reference matrix, as it just uses all the scRNA-seq data as the input. As such, this procedure requires substantial computational resources and time to handle the huge amount of the data. Although down-sampling can address this problem, scRNA-seq data usually suffer from the problem of extremely high dropout rate, especially for those generated by 10× Genomics. Thus, the reference matrix generated by down-sampling is often unhelpful for such considerations and will result in a poor deconvolution effect.

**Figure 1:**
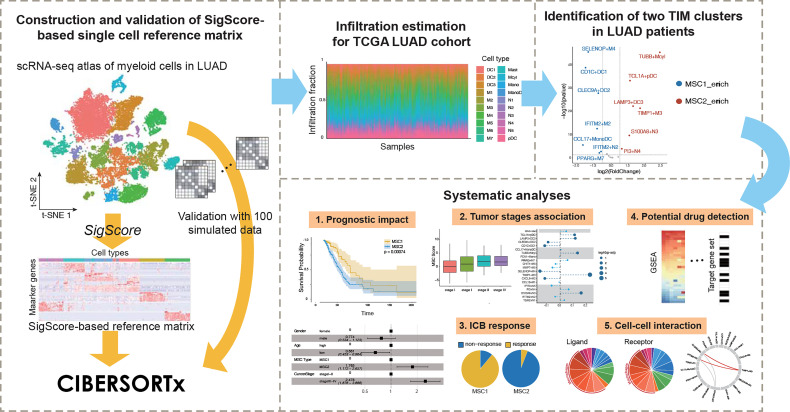
Conceptual view of study design.

In this paper, we applied a timesaving approach to create a customized reference matrix for scRNA-seq data for myeloid cells and made a deconvolution of The Cancer Genome Atlas (TCGA) LUAD cohort (**Figure 1**). As a result, we identified for the first time two different enrichment patterns (called MSC subtype hereafter). A series of analyses, including survival analysis and multivariable Cox regression analysis with clinical features, revealed MSC subtype to be robust prognostic factors. We reveal the relationship between MSC subtype and immune checkpoint blockade (ICB) therapy and identify three TIM subtypes that might contribute to the ICB response. Finally, we explore the heterogeneity of macrophages and detected a functional relationship between macrophage and neutrophil subtypes.

## Methods

### Data Source and Preprocessing

The single-cell transcriptome file of five LUAD patients and the validation data for DCs’ distribution were downloaded from the Gene Expression Omnibus (GEO) database under the accession numbers GSE127465 (14) and GSE131907 (23). The transcriptome expression profiles and corresponding clinical information for LUAD were retrieved from the Genomic Data Commons Data Portal of TCGA. Expression data were converted from counts type to transcripts per million (TPM). Three transcriptional microarray expression data (GSE matrix files) for LUAD cohorts [GSE31210 (24), and GSE72094 (25)] were obtained from the GEO. The microarray datasets were log-transformed (on a base 2 scale) and genes were detected with more than one probe retaining its maximum value.

### scRNA-seq Data and Single-Cell Data Analysis

The Seurat package (version 3.0) was used to perform scRNA-seq analysis (26). Transcriptomes with more than 300 total counts, <10,000 total counts, and <20% of counts coming from mitochondrial genes were retained for subsequent analysis. From the remaining cells, gene expression matrices were normalized to the total unique molecular identifier (UMI) counts per cell and were log-transformed (on a base 2 scale). Dimensionality reduction was performed with uniform manifold approximation and projection (UMAP). The marker gene of each cluster was identified using Seurat.

### Single Cell Reference Matrix Construction

The most characteristic cells in each cell subtype, rather than all tens of thousands of cell’s data, were selected to create the custom signature matrix. We performed a three-step approach to generate it. First, to utilize the gene expression signature and reduce the technological noise, we performed differential expression gene analysis by *FindMarkers()* function in Seurat and only retain those informational gene (log_2_ FC > 0.25) to construct the custom signature matrix. Second, we defined and calculated a so-called cell-type-specific signature score *SigScore* to select the candidate cells from each cell subtype.


$$SigScore=\sum_{i=1}^{N}\left([Marker_{i}]*k_{i}\right)$$

where [*Marker_i_*] represents the expression level of the marker gene, *i* and *k_i_* is the value equal to $log_{2}FC_{Marker_{i}}$ , which is calculated using the *FindMarkers*() function in Seurat. *N* denotes the number of the markers in each cell type. Note that *N* is variable according to the threshold of *log*_2_ *FC*. Specially, 1.25 was selected as the threshold of *log*_2_ *FC* in our paper.

We ranked all cells belonging to a special cell subtype in descending order of their *SigScore* and chose the top 50 cells to create the custom signature matrix. In addition, we created another custom signature matrix by randomly selecting 50 cells of each cell type, which would then be used as another matrix for comparison.

### Infiltration Estimation of the Myeloid Cells

As the last step of our approach, scRNA-seq data of the top 50 cells were uploaded to CIBERSORTx (http://cibersortx.stanford.edu) to create a customized signature matrix for each myeloid cell subtype by functional module “Create Signature Matrix” in CIBERSORTx. “Impute Cell Fraction” module in CIBERSORTx was used to quantify the infiltrating level of each myeloid cell subtype. A hundred simulated bulk datasets were created by random sampling of different numbers of each cell types (including non-myeloid cells) and were used to validate the signature matrix. Since count data were uploaded for creating the signature matrix, count-per-million (CPM) data of the TCGA LUAD cohort within the genes involved in the signature matrix were then generated to estimate the abundance of myeloid cell subtypes in CIBERSORTx.

### MSC Subtype Identification and Prediction Model Building

Based on the infiltration level of myeloid cell subtypes, the optimal number of TCGA LUAD cohort clusters were examined using the *mclust* package (version 5.4.5) (27). *K-Means* consensus clustering was conducted in R to determine distinct clusters of MSC subtypes. Hierarchical clustering was performed by *hclust()* in R, and the defined clusters were compared with the *K*-*Means* based clusters to ensure the robustness of the method we used. The least absolute shrinkage and selection operator (LASSO) algorithm was used to reduce the data dimensions and distinguish the most informative genes for predicting the MSC subtype using the glmnet package (version 3.0-2) (28, 29). Finally, the MSC score formula was calculated by considering the correlation estimated Cox regression coefficients:


$$M_{Score}=\sum_{i=1}^{Z}\left([Gene_{i}]\times coef_{i}\right)$$

where *Z* denotes the gene number determined by LASSO, [*Gene_i_*] denotes the expression level of gene *i*, and *coef_i_* represents the coefficient of gene *i* as determined by LASSO.

### Potential Drug-Targeted Gene Set Selection

To identify the potentially druggable therapeutic targets for the patients of identified MSC subtype, we collected two datasets, the genome-scale CRISPR knockout screens dataset in Project Achilles (https://depmap.org/portal/) and drug-induced gene expression profiles from the Library of Integrated Network-Based Cellular Signatures (LINCS; https://commonfund.nih.gov/LINCS/) L1000 dataset. We then performed a two-step analysis to identify candidate drugs. First, we filtered the essential genes for LUAD cell lines based on the genome-wide CRISPR gene essentiality scores (CERES) from Project Achilles. The genes whose CERES was lower than −0.5 in half of the total LUAD cell lines were retained and then intersected with the upregulated genes in MSC2 patients. We then ranked the gene expression profiles of LUAD cell lines obtained from LINCS and performed Gene Set Enrichment Analysis (GSEA) using the clusterProfiler package based on the above target gene set (30). Only when the target gene set was significantly enriched in the bottom of ranked gene list, the drug was then considered to have potential.

### Calculation of Ligand–Receptor Interaction

For the cell–cell interaction analysis, the expression level was normalized according to the total reads count and converted into a TPM-like scale. The expression values were averaged within each cell subtype. We retrieved the ligand–receptor pairs from a systematic research including known ligand–receptor pairs from the existing databases and predicted the ligand–receptor pairs with high confidence (31). The threshold of 1 TPM was used as the cut-off for ligand–receptor pairs within each cell subtype for further analysis.

### Statistical Analysis

The differentially expressed genes (DEGs) were calculated using the DESeq2 package for R (32). DEGs satisfying |log2 fold change| > 1.5 and adjusted p < 0.05 criteria were considered statistically significant. Clusterprofiler was used to perform Gene Ontology (GO) function enrichment and Kyoto Encyclopedia of Genes and Genomes (KEGG) pathway annotation. Within a specific cohort, patients were divided into two groups based on the mean value of *M_Score_* in all samples. Survival curves were constructed using the Kaplan–Meier (KM) method and compared using the log-rank test provided in the survival package for R (33). Multivariate Cox proportional hazard regression modeling was used to verify the prognostic significance for OS. Histological grade, gender, and age were used as variables. To identify the relationship between clinical state and myeloid cells, we queried the clinical data of the TCGA LUAD cohort. In particular, the tumor–node–metastasis (TMN) stages were categorized to a numeric level. The correlation between the infiltration level of myeloid cells and clinical variables was examined using Pearson’s correlation coefficient (CC), which was considered statistically significant by FDR < 0.05. Area under the curve (AUC) of the receiver operating characteristic (ROC) curve was used to assess the predictive ability of the predicted signature.

## Results

### Construction and Validation of *SigScore*-Based Reference Matrix

In recent researches and applications, purified-cell-based reference matrix has been widely used to perform deconvolution analysis. For example, LM22, a reference matrix generated by Newman et al. (18), distinguishes 22 human hematopoietic cell phenotypes, including seven T-cell types, naive and memory B cells, plasma cells, natural killer (NK) cells, and myeloid subsets. This matrix was employed to infer the infiltration level of above hematopoietic cells in bulk transcriptomic profiles. However, it leads to some limitations at the same time. First, cell transcriptome was tissue specific, so publicly available reference matrix cannot represent the real condition appropriately in different tissues. Besides, with the development of single cell sequencing, more and more researchers notice that there are still many functional subtypes even in one cell type (for example, M0 macrophages and M1 macrophages). This means that purified-cell-based matrix may not reflect the complexity of cellular compositions. Thus, we choose the scRNA-seq data to generated our reference matrix.

We downloaded the scRNA-seq data from Zilionis et al. (14) in which the authors demonstrated the major aspects of the lung tumor immune microenvironment. Twenty subtypes of myeloid cells were identified using Seurat (**Supplementary Figure 1A**). The corresponding scRNA-seq data of the top cells with the highest *SigScore* were then uploaded to CIBERSORTx, and the underlying reference matrix was obtained.

**Figure 2:**
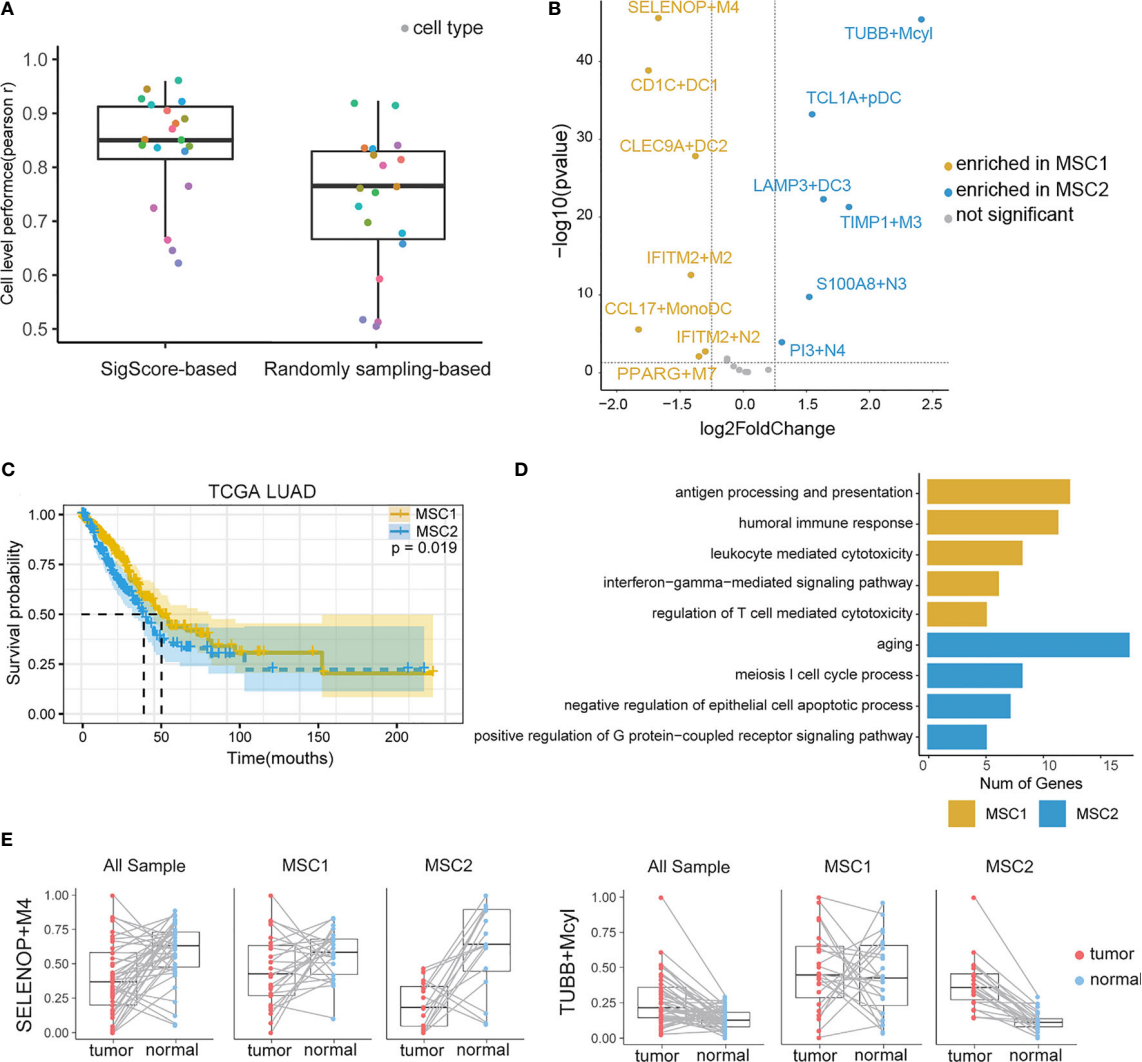
TIM infiltration landscape of myeloid cells in LUAD. **(A)** Performance comparison of two reference matrixes. **(B)** Different infiltration levels of myeloid cells between MSC1 and MSC2 patients. For brevity, myeloid cells were named as marker gene plus short name. M, N, DC, Mcyl, Mono, pDC, and MonoDC represent macrophages, neutrophils, dendritic cells, monocytes highly expressing cell-cycle-related gene, monocytes, plasmacytoid DCs, and one cell type showing the signatures of both monocytes and DCs, respectively. **(C)** KM plot for MSC subtypes in TCGA LUAD cohort. **(D)** GO functional annotation of MSC1 and MSC2. **(E)**Infiltration levels of two myeloid cell subtypes (SELENOP+M4 and TUBB+Mcyl) in tumor and tumor-adjacent tissues.

We examined the *SigScore*-based reference matrix compared to a randomly selected reference matrix within 100 simulated datasets. Our reference matrix showed better performance (**Figure 2A**).

### Identification of Two Distinct Myeloid Cell Infiltration Subtypes in LUAD Patients

We applied the *SigScore*-based reference matrix to investigate the fractions of infiltrated myeloid cells in the TCGA LUAD dataset (**Supplementary Figure 1B**). Among the total samples, 485 tumor samples were eligible for CIBERSORTx under *p* < 0.05 and CC > 0.5. We performed *K-means* clustering with the optimal number (*k* = 2) and identified two distinct myeloid cell infiltration clusters, namely, MSC1 and MSC2, according to the contextures of the myeloid cells (**Supplementary Figures 1C, D**). To evaluate the robustness of *K-Means* clustering, we performed hierarchical clustering, and we noted that those two methods showed high consistency (**Supplementary Figure 1E**, left). Meanwhile, random signature failed in the patient stratification (**Supplementary Figure 1E**, right). Thirteen of the 20 had distinct enrichment patterns between MSC1 and MSC2 (|log2 fold change| > 1.25, *p* < 0.05) (**Figure 2B**). MSC2 was significantly associated with a shorter OS compared with MSC1 (**Figure 2C**, *p =* 0.019). GO analysis suggested that the MCS1 subtype, with its favorable outcome, has a stronger immune response ability including both innate and adaptive immunity. Conversely, the MCS2 subtype was significantly associated with the cell cycle and negative regulation of cell apoptosis, in line with unfavorable outcomes (**Figure 2D**).

We queried the distribution of distinct TIM cell types within tumors and adjacent tissues. The statistical result is showed in (**Supplementary Table 1**). Some of those cell types are strongly related with OS. A high fraction of LAMP3+DC3, S100A8+N3, PI3+N4, TIMP1+M3, and TUBB+Mcyl were identified as poor prognostic factors, while high fraction of CLEC9A+DC2 were identified as a protective (**Supplementary Figure 2**).

We then observed that SELENOP+M4, the most enriched cell type in MSC1, was the subtype of macrophages that highly expressed SELENOP and TM4SF1 mRNAs. TUBB+Mcyl, the most enriched cell type in MSC2, was the subtype of the monocytes which highly expressed TUBB. The infiltration levels of two subtypes between the tumor and paired adjacent tissues of all patients, MSC1 patients and MSC2 patients, were further analyzed, respectively. SELENOP+M4 showed preferential enrichment in normal tissues. While considering the MSC subtype, we found a significant lower infiltration level of SELENOP+M4 in MCS2, while TUBB+Mcyl showed the opposite trends (**Figure 2E**). The infiltration levels of other cell subtypes in three states are shown in **Supplementary Figure 3**.

### Association of Predicted MSC Subtype With Prognostic Impact in TCGA and Two Independent LUAD Cohorts

**Figure 3:**
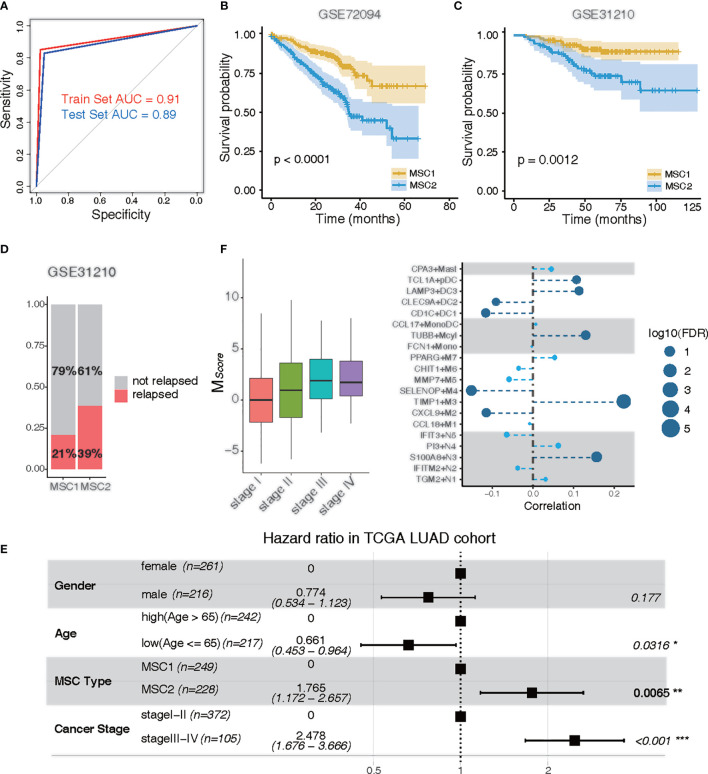
Clinical traits of MSC subtypes. **(A)** ROC curve of MSC subtype classifier in the training data and the test data. Training set, red; test set, blue. **(B, C)** KM plots for MSC types in GSE72094 **(B)** and in GSE31210 **(C)**. MSC1, yellow; MSC2, blue. **(D)** Rate of relapse in MSC1 and MSC2 patients in GSE31210. Patients with relapse (n = 28) and non-relapse (n = 105) in MSC1 type; patients with relapse (n = 36) and non-relapse (n =57) in MSC2 type. **(E)** Multivariable Cox proportional hazard regression analysis in TCGA LUAD cohort. **(F)** Correlation of tumor stages and the infiltration of myeloid cells in TCGA LUAD cohort. Left: MSC score in different tumor stages (stage I, n = 257; stage II, n = 115; stage III, n= 81; stage IV, n =24); Right: correlation between tumor stages and myeloid cell types. The size of the circle represents log10 (FDR); the dark blue circle indicates a significant correlation (FDR < 0.05).

To predict the MSC subtype for bulk RNA-seq or microarray data, we determined the most informative genes and constructed a resulting 14-gene signature (i. e.,*M_Score_*) (**Supplementary Table 2**). The TCGA LUAD cohort were randomly divided into a training set (n = 292) and a test set (n = 193). The LASSO Cox regression model with 20-fold cross-validation was performed to train the model in the training set. We then assessed the model performance in the test set. According to the *M_Score_*, LUAD patients were well classified into MSC1 and MSC2 subtypes. The AUC of the ROC curve achieved 0.91 and 0.89 in the training and test sets, respectively, indicating a strong prediction ability to stratify the patients (**Figure 3A**).

The prediction capability of 14-gene signature was executed to further examine the prognostic significance in two independent LUAD microarray cohorts (GEO72094 and GEO31210). Similarly, a poor prognostic impact of MSC1 compared with MSC2 was found in both cohorts (*p <* 0.0001 and *p =* 0.0012, respectively) (**Figures 3B, C**). We also observed that nearly twice the relapse rate had occurred in MSC2 compared with MSC1 in the GSE31210 dataset. This suggested that the myeloid cell distribution might be associated with cancer relapse (**Figure 3D**).

To determine whether MSC subtype is an independent predictor of the prognosis, we performed multivariate analysis. In the TCGA LUAD and GSE72094 cohorts, MSC2 strongly predicted a shorter OS compared with MSC1, independent of known risk factors [hazard ratio (HR), 1.765; 95% CI, 1.172–2.567; *p* = 0.0065 for TCGA; HR, 2.680; 95% CI, 1.789–4.020; *p* < 0.001 for GSE72094), including age, gender, and cancer stage (**Figure 3E**; **Supplementary Figure 4A**).

### MSC Subtype Significantly Associated With Tumor Stages of LUAD

We analyzed the distribution of *M_Score_* in different tumor and TNM stages. The higher *M_Score_* was associated with a higher tumor stage (**Figure 3F**). We also observed a similar positive correlation in both T, M, and N stages, suggesting that the distribution of myeloid cells were potentially related to the clinical stage (**Supplementary Figures 4B**–**D**). To further investigate whether the relative presence of these myeloid cell subtypes was associated with tumor progression, we calculated the correlation between each myeloid cell subtype and cancer stage. Five cell types, TIMP1+M3, S100A8+N3, TUBB+Mcyl, LAMP3+DC3, and TCL1A+pDC, all of which were enriched in MSC2, were positively associated with tumor progression (FDR < 0.05). Four cell types, SELENOP+M4, CXCL9+M2, CD1C+DC1, and CLEC9A+DC2, were negatively associated with tumor progression (FDR < 0.05) (**Figure 3F**).

### MSC Subtype Associated With Immunotherapy Response

**Figure 4:**
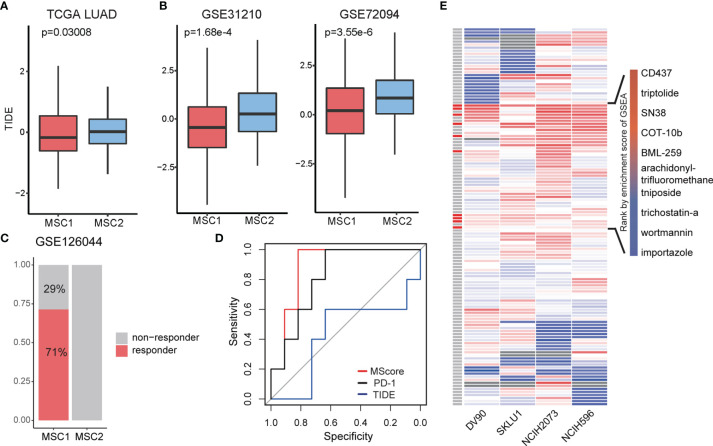
Exploration of treatment for two MSC type. **(A, B)** TIDE score in different MSC subtypes for TCGA LUAD and two independent cohorts. **(C)** Rate of ICB response in MSC1 and MSC2 patients in GSE126044. Patients with response (n = 5) and non-response (n = 2) in MSC1 type; patients with relapse (n = 0) and non-relapse (n = 9) in MSC2 type. **(D)** ROC curve of ICB response prediction. *M_Score_*, red; expression of PD-1, black; TIDE score, blue. **(E)** Enrichment score for each drug in four cell lines. On the left most column, red box indicates the drug has therapeutic potential in total four cell lines, which are listed on the right side.

Myeloid cells have been reported to be associated with ICB (34). In the TCGA LUAD cohort, we introduced the tumor immune dysfunction and exclusion (TIDE) algorithm to explore the relationship between the MSC subtype and ICB response (35). TIDE is a computational framework for predicting the clinical response to ICB in patients. A low TIDE prediction score indicates that the patients would potentially exhibit a greater immune therapy response. We observed the TIDE score as significantly lower in MSC1, suggesting that the MSC1 is more likely to respond to ICB therapy (Wilcoxon rank-sum test, *p* < 0.05) (**Figure 4A**). This association was verified in two independent cohorts using univariate analysis (Wilcoxon rank-sum test, *p* < 0.05) (**Figure 4B**). In the other independent cohort (i.e., GSE126044), which includes the RNA-seq data and response states of 16 patients before antiprogrammed cell death protein 1 (PD-1) treatment, we compared the TIDE and *M_Score_* to evaluate the prediction performance of ICB response. All five responders of 16 patients were clearly identified as MSC1 (**Figure 4C**). *M_Score_* showed the best predictive power (AUC = 0.891) as compared to TIDE and PD-1 (**Figure 4D**).

### Identification of Potential Drug for MSC2 Patients

Since MSC2 patients seem more unlikely to respond to ICB therapy and show a worse survival state compared with MSC1 patients, we next focused on identifying the potential drugs for patients of the MSC2 subtype. The upregulated DEGs in MSC2 patients are supposed to be the therapeutic target. Note that MSC subtypes are classified according to the marker genes of myeloid cells, leading us to query whether the DEGs represent the diversity of tumor microenvironment. Using a hypergeometric test, we found that the myeloid cell markers were almost irrelevant with DEGs between MSC1 and MSC2 patients (**Supplementary Figure 5A**). This suggests that the DEGs might reflect differences in tumor cell state.

We screened the data of lung adenocarcinoma cell lines in Project Achilles (https://depmap.org/portal/). The genes with a CERES lower than −0.5 in half of the lung adenocarcinoma cell lines were retained. We then intersected the DEGs, which were upregulated in MSC2 patients with survival-related genes and obtained 29 genes that represent the targets for ICB therapy in MSC2 patients (**Supplementary Figures 5B, C**).

Eight of 29 genes were related to the cell cycle, suggesting that the tumor cells in MSC2 patients might be in a relatively strong state of cellular proliferation. The remaining genes were involved in a wide range of cancer-related pathways, such as DNA replication, Ras signaling, and mTOR signaling (**Supplementary Figure 5C**). Twenty-eight of 29 genes have been reported to be related with LUAD (36–40). However, TOPBP1 interacting checkpoint and replication regulator (*TICRR*) had only been previously reported to be important in DNA replication (41). *TICRR* seems to be a promising therapeutic target for LUAD, especially in MSC2 patients.

Since the potential drug target gene set was obtained, we employed LINCS L1000 dataset to identify potential drugs. We focused on four LUAD cell lines, DV90, SKLU1, NCIH2073, and NCIH596. Testing was conducted of 361 drugs on the four cell lines, with a total of 1,498 gene expression profiles extracted. After computing the robust z-scores for each profile relative to control, we ranked the gene based on the expression levels and performed GSEA analysis. We totally identified 129 drugs showing potential inhibition effect for at least a particular cell line (FDR < 0.05, **Figure 4E**). To obtain more reliable drugs, we selected the drugs that showed significant suppression effects on all four cell lines and discovered 10 drug candidates (**Figure 4E**).

### Identification of New Myeloid Cell Subtypes Related With ICB Response

**Figure 5:**
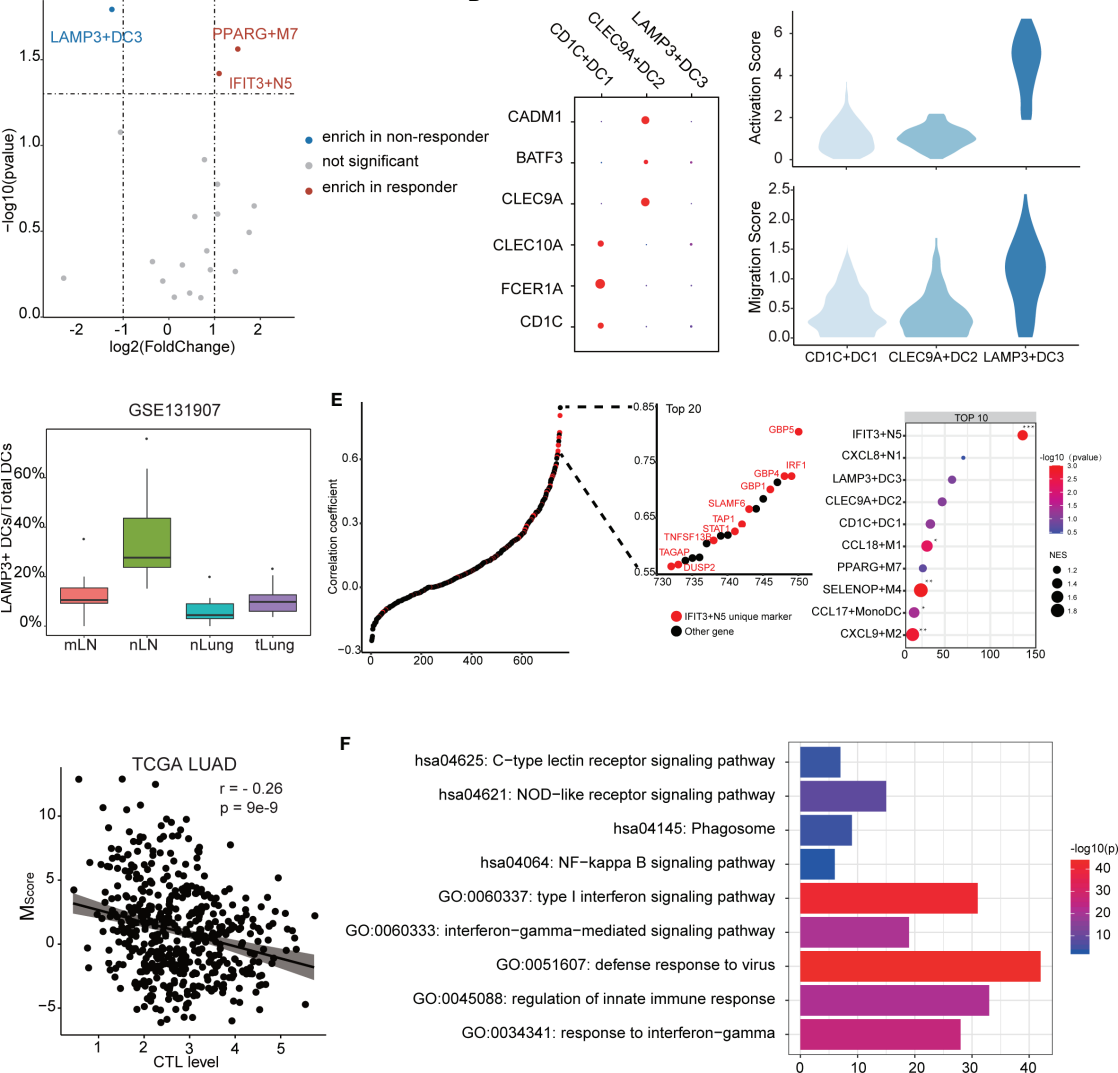
Characteristics of the myeloid cell subtypes related to ICB response. **(A)** Differences of myeloid cell infiltration levels between ICB responders and non-responders. **(B)** Transcriptome traits of dendritic cells. Activation and migration signatures are shown on the right. **(C)** Ratio of LAMP3+DCs of total DCs. mLN, nLN, nLung, and tLung denote metastatic, normal lymph nodes, normal lung tissues, and lung tumor tissues, respectively. **(D)** Correlation between *M_Score_* and CTL level. **(E)** Correlation of myeloid cell signature and CTL level. Marker genes were ranked based on the Pearson’s correlation coefficient. Left, the marker genes of IFIT3+N5 are denoted in red. Top 10 enriched marker gene sets of myeloid cells as identified by GSEA analysis. **(F)** GO and KEGG functional annotation of IFIT3+N5.

To further understand which myeloid cell subtype contributed to ICB response, we further investigated the fractions of infiltrated myeloid cells in GSE126044 dataset by applying CIBERSORTx. We observed that IFIT3+N5 and PPARG+M7 were enriched in responders, while LAMP3+DC3 was the only subtype enriched in non-responders (**Figure 5A**). CD1C+DC1 highly expressed CD1C, FCER1A, and CLEC10A, corresponding to conventional cDC2, while CLEC9A+DC2 highly expressed CLEC9A, BATF3, and CADM1, corresponding to conventional cDC1 (42, 43) (**Figure 5B**).

According to existing knowledge, there is no LAMP3+DC3 counterpart in the classic DC subsets; we thus compared transcript profiles among three DC subtypes. We observed that LAMP3+DC3 highly expressed an “activated” DC signature in line with the previous study (16) and showed a higher migration ability, according a gene signature derived from mouse tissue-migratory cDCs (**Figure 5B**) (44). We then compared the ratio of LAMP3+ DCs/total DCs in GSE131907 dataset (23). The result showed that LAMP3+DCs were enriched in the lymph nodes in LUAD patients, which further demonstrates its migration ability (**Figure 5C**). Interestingly, GO analysis showed that LAMP3+DC3 was associated with negative regulation of the immune system (**Supplementary Figure 6A**), which seems to explain its enrichment in non-responders. We also note that CD274 (PD-L1) was highly expressed in LAMP3+DC3 (**Supplementary Figure 6B**). Altogether, LAMP3+DC3 might play an important role in immunosuppression, especially in T-cell dysfunction, albeit more mature (16).

The degree of cytotoxic T-cell infiltration (CTL) has been reported to influence ICB effectiveness and has been used as a parameter of TIDE (35, 45). We used the average expression of PRF1, GZMA, GZMB, CD8A, and CD8B to estimate the CTL levels and examined the correlation between *M_Score_* and CTL levels. Interestingly, a significant but moderate negative correlation was found (*r* = −0.26, *p* = 9e−9), suggesting that myeloid cells might affect the CTL level (**Figure 5D**). It could be considered that the genes with high correlation with CTL level not only exist as a biomarkers but also as a clue of potential useful cell subtype. Notably, the unique marker gene of IFIT3+N5 was enriched in the gene set, which highly correlated with CTL level (**Figure 5E**). Ten of the top 20 highly correlated genes were unique markers of IFIT3+N5. IFIT3+N5 was found to be involved in the viral defense response, response to INF-gamma, and type I interferon signaling pathway. KEGG pathway analysis showed that IFIT3+N5 was strongly associated with NOD-like receptor signaling pathway and NF-kappa B signaling pathway (**Figure 5F**). We noted that three guanylate-binding family proteins (GBP5, GBP4, and GBP1) in the top 20 correlated genes had been identified to be linked to the inflammasome activation (46, 47) and were also highly correlated with CTL level (0.804, 0.725, and 0.701). Altogether, IFIT3+N5 might play a role in activate the CD8+ cytotoxic cell response in LUAD, which act, in part, through inflammasome activation.

### TIMP1+M3 Macrophages Recruit S100A8+ Neutrophils *via* CXCL5–CXCR2 Axes to Promote LUAD Progression

**Figure 6:**
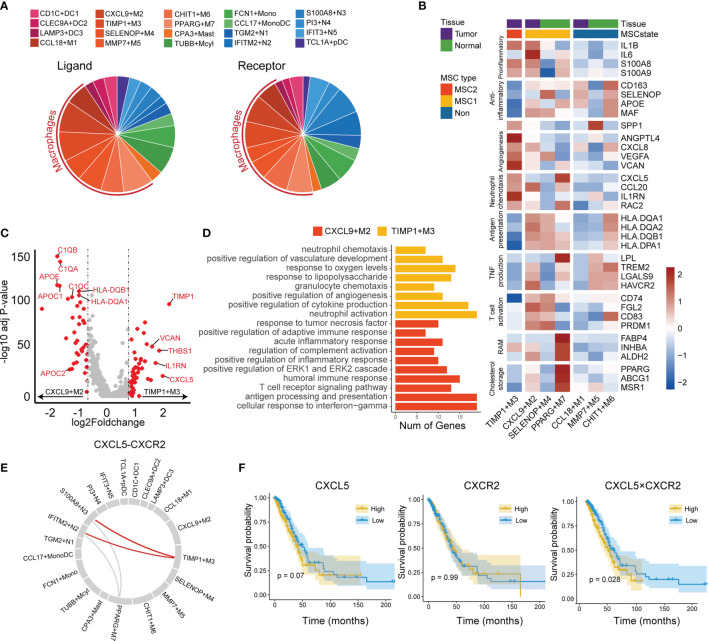
Tumor-associated macrophages in tumor microenvironment. **(A)** Cell communication in LUAD immune microenvironment. The ligand (left) is displayed separately from the receptor (right). **(B)** Functional gene expressions in each macrophage subtypes. Tissue and MSC subtype enrichment state is shown as annotations. **(C)** Differentially expressed genes between CXCL9+M2 and TIMP3+M3 subtypes. Top 20 genes in each macrophage subtype were labeled. **(D)** GO functional annotation of CXCL9+M2 and TIMP3+M3. **(E)** Circos plot for predicted interactions mediated by CXCL5-CXCR2. **(F)** KM plots for TCGA LUAD cohort stratified by the expression levels of CXCL5 and CXCR2 and the product of CXCL5 and CXCR2.

We used predefined ligand–receptor pairs (31) to examine the interactions between the myeloid cells. In terms of cell communications, we observed that macrophages showed higher proportions than other cell types (51% in ligand and 45% in receptor), suggesting macrophages may act as a hub for other myeloid cells (**Figure 6A**).

The macrophage subtypes included both proinflammatory (M1), anti-inflammatory (M2), and a mixed phenotype, suggesting that the M1 and M2 types are underestimates of tumor-associated macrophage complexity (**Figure 6B**). CCL18+M1, CXCL9+M2, and TIMP1+M3 were enriched in tumor tissues and were regarded as TAMs, whereas SELENOP+M4, MMP7+M5, CHIT1+M6, and PPARG+M7 were enriched in normal tissues and considered as resident tissue macrophages (RTMs) (**Supplementary Figure 7A**).

We then focused on the subtypes enriched in either MSC1 or MSC2 type. TIMP1+M3 was the unique subtype enriched in MSC2, suggesting its potential ability to promote tumor progression. CXCL9+M2, the subtype also enriched in tumor tissues, showed an opposite enrichment pattern. By further comparing the RNA expression profiles between two TAM subtypes (i.e., TIMP1+M3 and CXCL9+M2), we observed that CXCL9+M2 exhibited a high expression of C1Q family genes, apolipoprotein family genes, and antigen-presentation-related genes (**Figure 6C**). In contrast, TIMP1+M3 showed specific expression of TIMP1, VCAN, and CXCL5. GO analysis revealed a strong enrichment of complement activation, immune response, and antigen processing and presentation pathway in CXCL9+M2, while positive regulation of angiogenesis and neutrophil chemotaxis showed significant enrichment in TIMP1+M3 (**Figure 6D**). Meanwhile, we found that TIMP1+M3 showed a functional relationship with neutrophil, as indicated by GO annotation. By the cell–cell interaction analysis, TIMP1+M3 was predicted to interact with neutrophils (IFITM2+N2 and S100A8+N3) *via* CXCL5–CXCR2 axes, suggesting that TIMP3+M3 attracted the neutrophil *via* the chemokine (**Figure 6E**). S100A8+N3, which was attracted by TIMP1+M3 *via* the CXCL5–CXCR2 axes, was identified as a risk factor and showed moderate positive correlation with TIMP3+M3, while its correlation with IFITM2+N2 was negative (**Supplementary Figures 2** and **7B**). We also observed the higher value CXCL5 × CXCR2 was associated with an unfavorable OS, whereas no such association was observed for either CXCL5 or CXCR2 (**Figure 6F**). Interestingly, TIMP1+M3 and S100A8+N3 were positively correlated with N stage, suggesting their important role in lymph node metastasis (**Supplementary Figure 4C**).

We also investigated other macrophage subtypes. SELENOP+M4 showed high expressions of CCL4L2, CCL3L3, CCL3, and CLL4. GO analysis revealed leukocyte chemotaxis and positive regulation of cytokine production pathway as enriched in SELENOP+M4 (**Supplementary Figure 7C**). SELENOP+M4 also highly expressed antigen presentation and T-cell activation-related genes, suggesting it might play an important anticancer role and involvement in immune activation (**Figure 6B**). PPARG+M7 was identified as resident alveolar macrophage with high expressions of PPARG, FABP4, INHBA, and ALDH2 (48). We noted that PPARG+M7 was enriched in ICB responders, suggesting that it may also play a key role in regulating antitumor immunity as a tissue-specific macrophage.

## Discussion

We generated a TIM-related genes-specific reference matrix based on scRNA-seq data set and calculated the infiltration of TIM subtypes in the TCGA LUAD cohort. According to intratumor TIM heterogeneity, patients were stratified into two groups, MSC1 and MSC2. We found that MSC subtype was strongly associated with OS and ICB responses. Specific TIM subtypes showed particular functions in tumor progression. In discussing this work, we focused on the results that have promising applications and those that are closely related to clinical treatment.

We proved that the MSC subtype represents the states that either may or may not respond to ICB therapy in multiple datasets. In particular, the MSC subtype was useful for the estimation of differences in TIMs infiltration states. We validated the effectiveness of MSC subtype by comparing it with the TIDE score, which mainly considered the function of cytotoxic T cells as predictive of ICB response. The TIDE score shows significant differences within two MSC subtypes in TCGA and in the two GEO datasets. Since both lymphocytes and myeloid cells have been reported to be related with ICB response (49), we believe that MSC subtypes and TIDE score reflected different aspects of ICB responses of patients and that MSC subtypes could be jointly used with TIDE score to achieve a better estimations in a clinical context.

In our paper, three TIM subtypes were identified as ICB response related, including LAMP3+DC3, IFIT3+N5, and PPARG+M7. LAMP3+DC3 was enriched in ICB nonresponders and was identified as a more mature DC subtype. Compared with other two DC subtypes, CD1C+DC1 and CLEC9A+DC2, the negative regulation of immune system process pathways were enriched in LAMP3+DC3. CCR7 is necessary for the migration of tumor-infiltrating DCs into tumor-draining lymph nodes (50). LAMP3+DC3 highly expressed CCR7 and showed the strongest migration ability, indicting LAMP3+DC3 might migrate to the lymph node and suppress immune activation. Similar DCs were identified in the single cell study of hepatocellular carcinomas and were described to be related with T-cell dysfunction by interacting with T lymphocytes (16). Zhang *et al.* also suggested that LAMP3+DCs in tumors might originate from cDC1 and cDC2. Thus, LAMP3+ DCs may not only be a predictive factor of ICB response but also could be considered as a new target for immunotherapy.

As multiple studies have demonstrated that tumor infiltrating neutrophils are related to cytotoxic T cells in various ways (51–53), we confirmed that our identified IFIT3+N5, a subtype of neutrophil, was enriched in ICB responders and showed positive correlation with CTL. Functional annotation indicated that IFIT3+N5 might activate CD8+ T lymphocytes, partly *via* inflammasome activation. According to a previous study, IFIT3+N5 corresponds to a group of mature neutrophils that are expanded in virus-infected tissues (54). Considering that neutrophils might be converted into different phenotypes, either anti- or protumoral (55), the clarification of how the precursor cells are changed to IFIT3+N5 will be an important consideration for future studies.

It has been reported that CXCR2+ neutrophils are recruited by CXCL5 in tumor tissues to promote tumor progression in liver and non-small cell lung cancers (56, 57). However, most of these studies used cell lines, tissue sections, and mouse models, which made it difficult to identify the specific cell subtypes involved. In this article, we clearly identified that TIMP3+M3 recruited S100A8+N3 *via* CXCL5–CXCR2 axes. TIMP3+M3 and S100A8+N3 were both identified as protumoral cell types and related with lymph node metastasis, suggesting that those cells might promote the tumor progression in synergy. CXCR2 and CXCR4 were seen as required when neutrophils egress from the bone marrow and are retained in the lungs (58). Here, we noticed a repulsive expression pattern between CXCR2 and CXCR4 in the neutrophils (**Supplementary Figure 7D**). Thus, a blockade of CXCR2 might lead to decreasing infiltration of S100A8+N3, which might partly explain the high performance of CXCR2 antagonists (59).

In summary, the main purpose of this paper was to develop a time-saving approach to quantify the cell-type proportions from bulk RNA-seq data at single-cell resolution. We generated the landscape of myeloid cells in LUAD and stratified the patients into two infiltrating patterns (MSC1 and MSC2). We observed a significant relationship between TIM infiltrating pattern and OS and ICB responses and validated this finding in two external independent cohorts. We identified special myeloid subtypes related with tumor progression and ICB response, leading to new insights into the function of TIMs in cancer. These findings could assist scientists in understanding the complexity of TIMs and help optimize related immunotherapy strategies. As the future work, functional studies, like immunophenotyping, are needed to clarify the special role of mentioned myeloid cells and their function in cancer immunotherapy.

## Data Availability Statement

Publicly available datasets were analyzed in this study, these can be found from the TCGA LUAD and the NCBI Gene Expression Omnibus under accession number: GSE127465, GSE131907, GSE31210, and GSE72094.

## Author Contributions

HW and CL conceived the basic idea. HW, JQ, and QZ further analyzed scRNA-seq and bulk RNA-seq data of LUAD. HW, QZ, and LL evaluated the results and validated the performance of the new approach. HW wrote the draft of the manuscript. JQ revised the whole contents. CL supervised the whole study. All authors contributed to the article and approved the submitted version.

## Funding

This study was supported by the Chinese National Natural Science Foundation (grant 82171939).

## Conflict of Interest

The authors declare that the research was conducted in the absence of any commercial or financial relationships that could be construed as a potential conflict of interest.

## Publisher’s Note

All claims expressed in this article are solely those of the authors and do not necessarily represent those of their affiliated organizations, or those of the publisher, the editors and the reviewers. Any product that may be evaluated in this article, or claim that may be made by its manufacturer, is not guaranteed or endorsed by the publisher.

## Abbreviations

AUC, area under the curve; CERES, genome-wide CRISPR gene essentiality scores; CTL, cytotoxic T-cell infiltration; DC, dendritic cell; DEG, differentially expressed genes; ICB, immune blockade therapy; LASSO, least absolute shrinkage and selection operator; LN, lymph node; LUAD, lung adenocarcinoma; OS, overall survival; RTM, resident tissue macrophages; scRNA-seq, single-cell RNA sequencing; TAM, tumor-associated macrophage; TIM, tumor infiltrating myeloid cell; TME, tumor microenvironment; TPM, transcripts per million; UMAP, uniform manifold approximation and projection; UMI, unique molecular identifier.
