# Supplementary material for: Microdissection of the Bulk Transcriptome at Single-Cell Resolution Reveals Clinical Significance and Myeloid Cells Heterogeneity in Lung Adenocarcinoma
Source: Front Immunol. 2021 Sep 30;12:723908. doi: 10.3389/fimmu.2021.723908 (PMC8515901; doi:10.3389/fimmu.2021.723908)
Supplement: Supplementary file 1 [file DataSheet_1.pdf]

Table

Table S1. Infiltration levels of myeloid cell types in tumor and paired normal tissues of TCGA LUAD cohort.

Cell type	Cancer	Normal	pvalue	Logfolechange	padj
PPARG+M7	0.1119	0.0178	3.89E-15	-2.66	7.79E-14
IFITM2+N2	0.0471	0.0199	5.03E-03	-1.25	8.38E-03
PI3+N4	0.0447	0.0247	1.75E-07	-0.85	1.17E-06
SELENOP+M4	0.0927	0.0529	1.97E-05	-0.81	5.63E-05
CPA3+Mast	0.0721	0.0433	1.69E-04	-0.73	3.76E-04
MMP7+M5	0.0858	0.0521	2.25E-06	-0.72	9.00E-06
LAMP3+DC3	0.0433	0.0304	8.97E-05	-0.51	2.24E-04
CHIT1+M6	0.0450	0.0354	1.13E-01	-0.35	1.51E-01
TCL1A+pDC	0.0542	0.0517	2.94E-01	-0.07	3.67E-01
VCAN+Mono	0.0395	0.0383	5.14E-01	-0.04	5.41E-01
CLEC9A+DC2	0.0406	0.0459	3.31E-01	0.18	3.89E-01
CD1C+DC1	0.0239	0.0334	6.41E-01	0.48	6.41E-01
S100A8+N3	0.0190	0.0273	2.08E-02	0.52	2.97E-02
CCL18+M1	0.0087	0.0148	4.32E-04	0.77	7.85E-04
IFIT3+N5	0.0097	0.0181	2.77E-04	0.90	5.55E-04
CCL17+MonoDC	0.0029	0.0057	3.57E-01	0.99	3.97E-01
TUBB+Mcyl	0.0435	0.1091	6.43E-07	1.33	3.22E-06
CXCL9+M2	0.0236	0.0746	3.01E-06	1.66	1.00E-05
TIMP1+M3	0.0181	0.0708	2.89E-10	1.97	2.89E-09
CXCL8+N1	0.0000	0.0009	6.59E-03	Inf	1.01E-02

Table S2. Coefficients of 14-gene signature.

gene_symbol	coefficients
CCL17	-0.007887324
CCR7	-0.141747905
CKAP4	0.114105821
H3F3A	0.124654023
HLA-DPA1	-0.588515534
HLA-DPB1	-1.061542723
IRF4	-0.00987777
JOSD1	0.0529529
LAD1	0.224285872
RNASE1	-0.141470851
RRM2	0.57193537
SOX4	0.098623767
TUBB	0.263887911
TXN	0.056643707

Figure

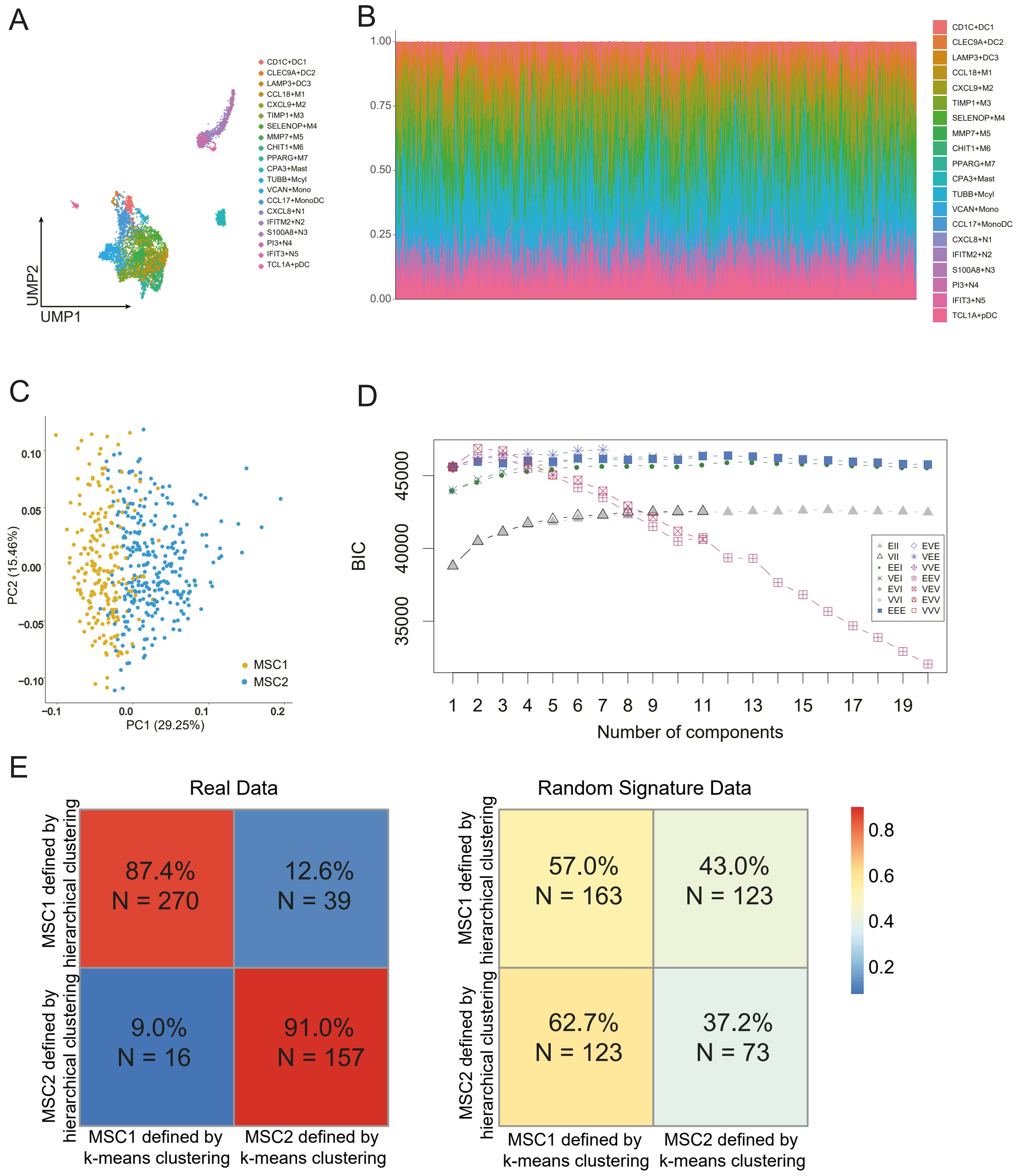

Figure S1. Construction of MSC subtypes. A. UMAP plot for myeloid cells in LUAD patients. **B.** Infiltrating fractions of myeloid immune cells in 485 LUAD patients. X-axis denotes the samples. Y-axis denotes the infiltrating fraction. **C.** Dot plot for principal co--mponent analysis of TCGA LUAD patients. The MSC type is labeled. **D.** Bayesian Info--rmation Criterion (BIC) score of different models and different cluster number. **E.** Heat--map for evaluating the robustness of *K-Means* clustering. The accuracy was represented by color.

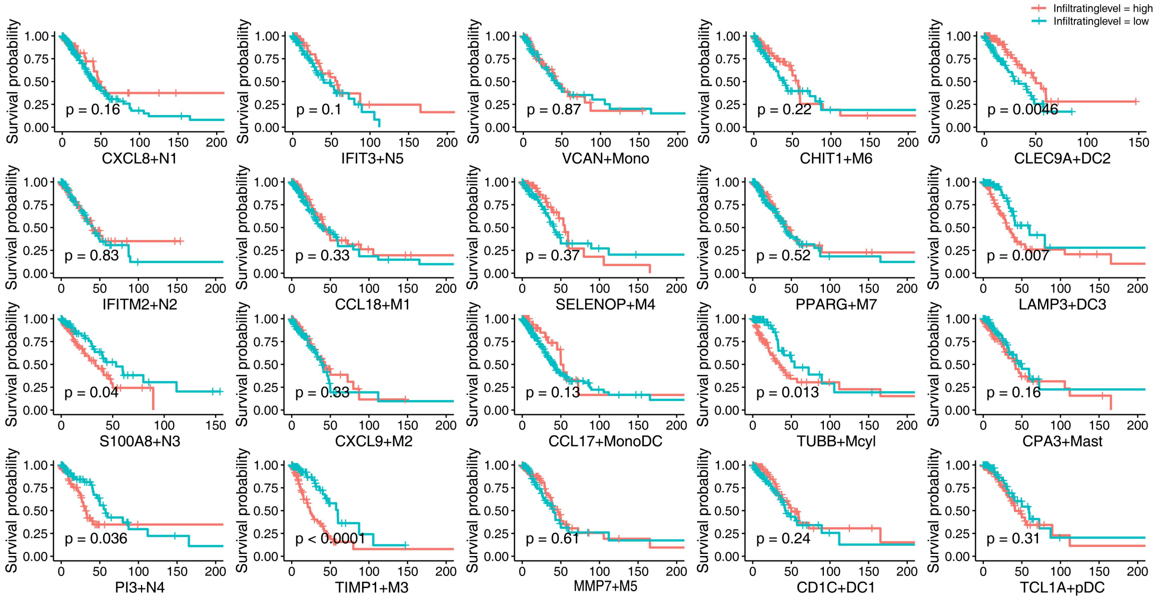

Figure S2. KM plots for TCGA LUAD cohort stratified by the infiltrating levels of myeloid cell types.

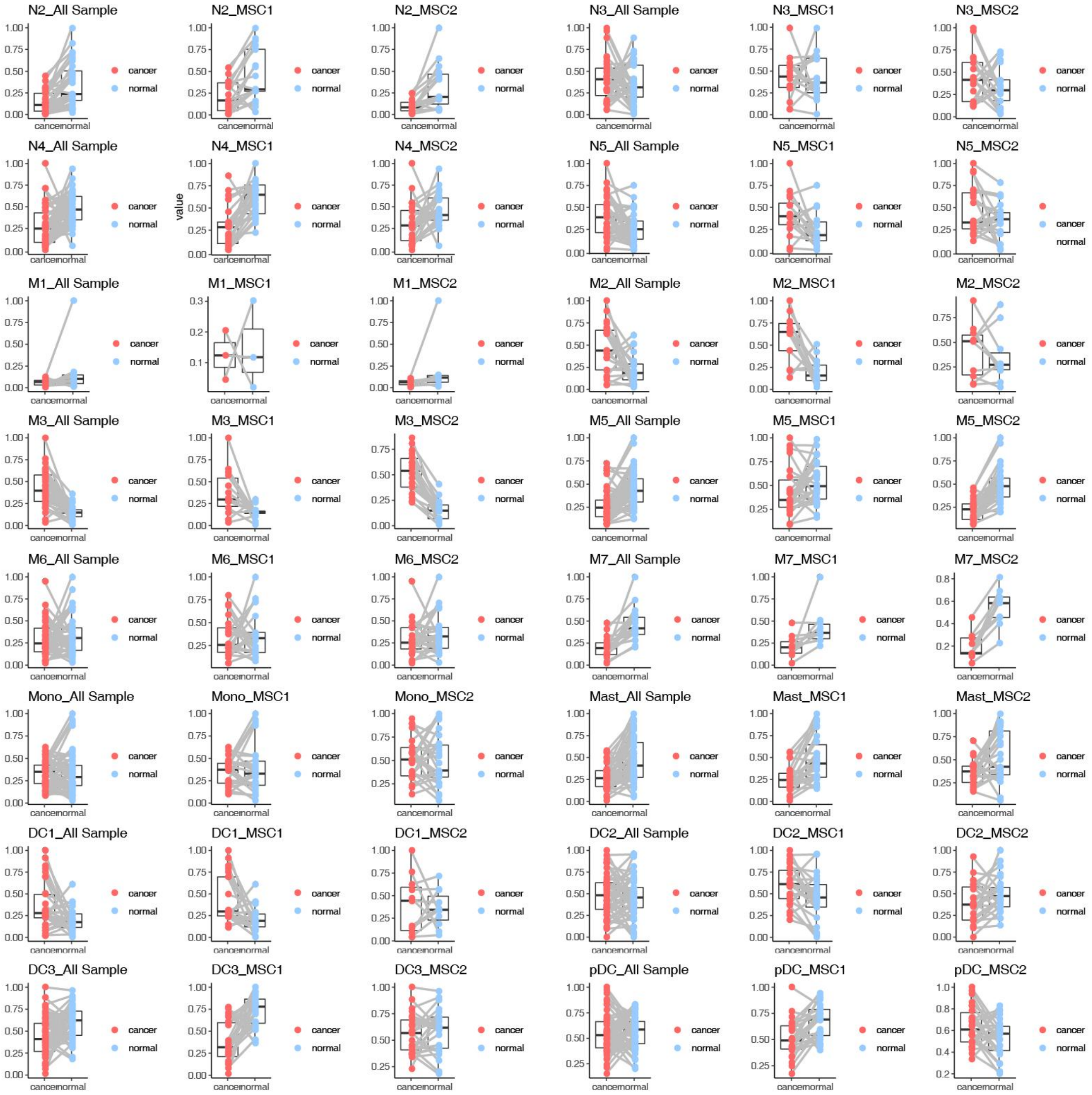

Figure S3. Paired box plot for the infiltrating levels of myeloid cell types in tumor and tumor-adjacent tissues.

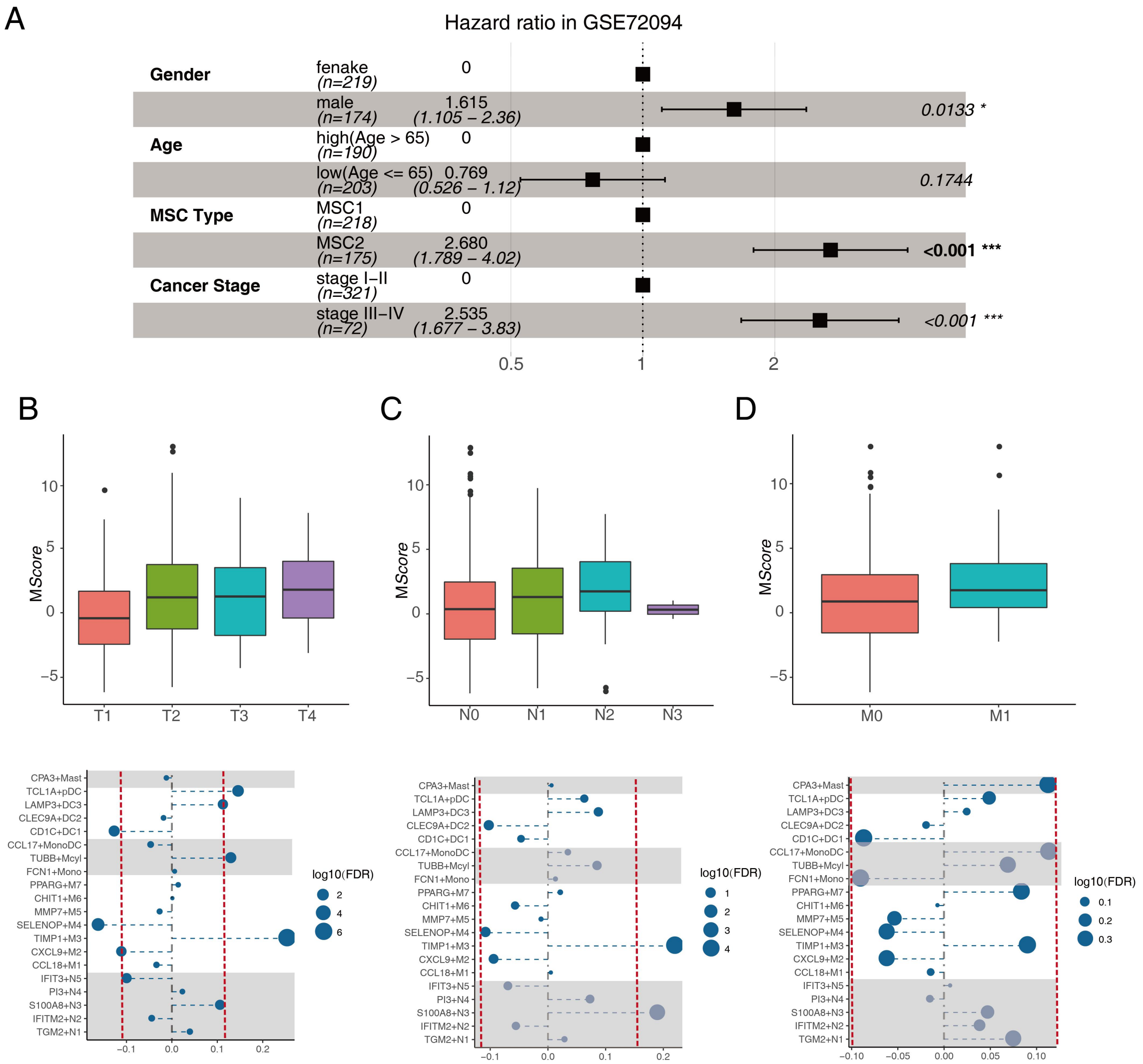

Figure S4. Clinical traits of MSC subtypes. A. Multivariable Cox proportional hazard regression analysis in GSE72094 dataset. **B-D.** Correlation between the myeloid cell type infiltration levels. **B.** T stage; **C.** N stage; **D.** M stage in TCGA LUAD cohort. The red line indicates a significant correlation with the threshold of FDR < 0.05.

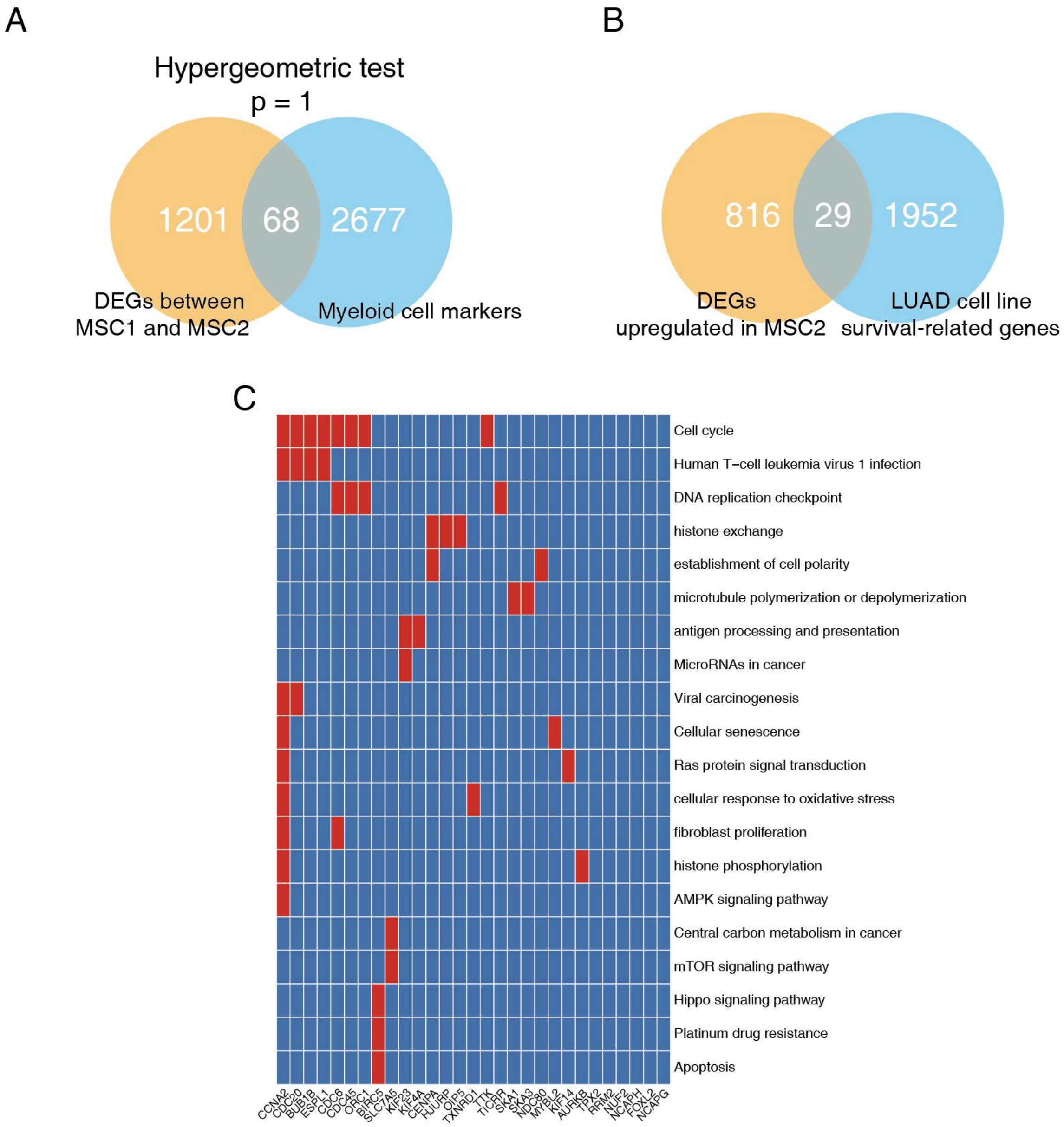

Figure S5. Potential drug targeted gene set of MSC2 patients. A. Overlap of DEGs between MSC1 and MSC2 patients and myeloid cell markers. **B.** Overlap of upregulated genes in MSC2 patients and survival-related genes in LUAD cell lines. **C.** Heatmap of functional annotation for therapeutic target of MSC2 patients. The red box indicates the gene is related to the pathway showed at the right-side.

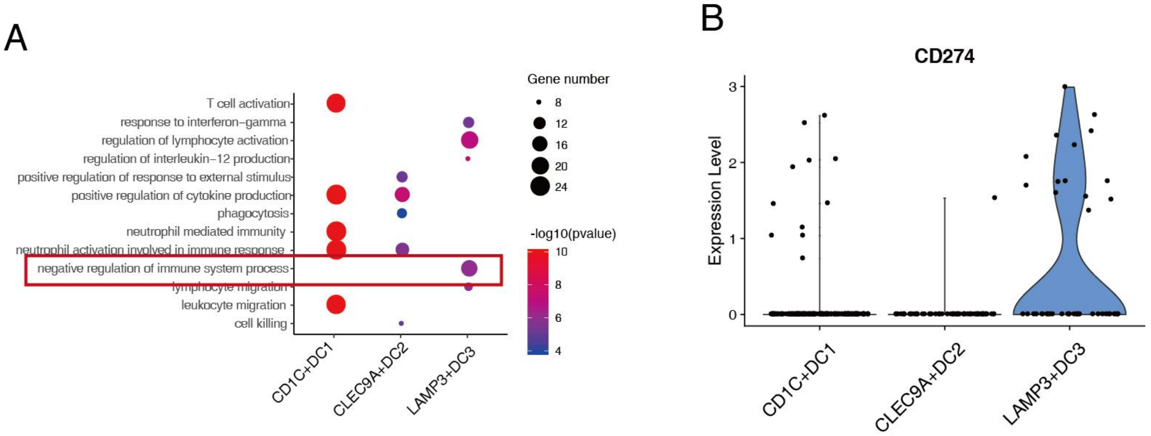

Figure S6. Transcriptomic characteristics of dendritic cell subtypes. A. Dot plot for GO functional annotation of dendritic cell subtypes. **B.** Violin plot for CD274 (PD-L1) expression level in dendritic cell subtypes.

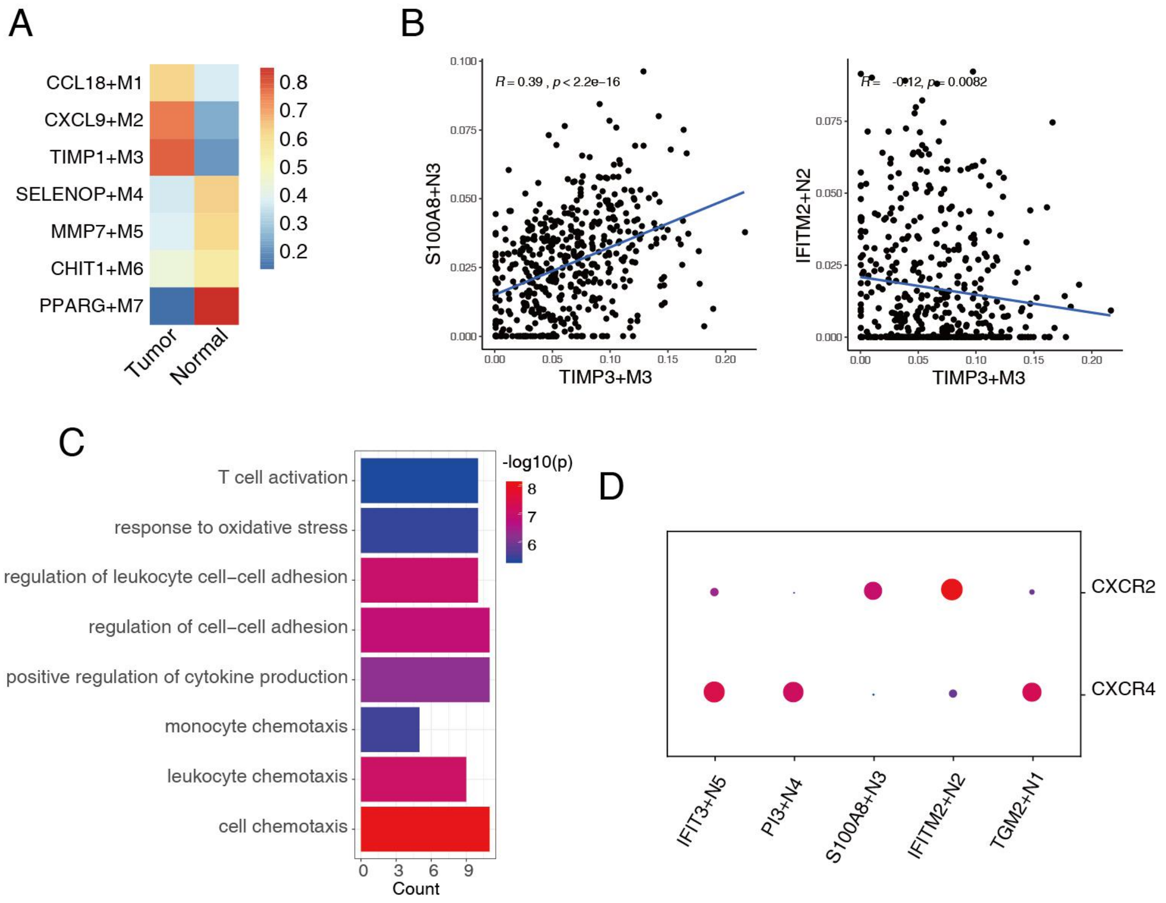

Figure S7. Macrophage-related transcription characteristics. A. Heatmap for tissue prevalence estimated by macrophage subtype infiltrating level. **B.** Scatter plot for the correlation of TIMP3+M3 with S100A8+N3 (left) and IFITM2+N2 (right) respectively. **C.** GO functional annotation of SELENOP+M4. **D.** The expression levels of CXCR2 and CXCR4 in neutrophils.
